# Identifying Womens' Needs in Making a Treatment Decision for Stress Urinary Incontinence: A Qualitative Study

**DOI:** 10.1089/whr.2023.0002

**Published:** 2023-07-18

**Authors:** Maria B.E. Gerritse, Ellis de Swart, Marieke de Vries, Kirsten B. Kluivers

**Affiliations:** ^1^ Department of Gynecology, Radboud University Medical Center, Nijmegen, the Netherlands.; ^2^ Department of Obstetrics and Gynecology, Gelderse Vallei Hospital, Ede, the Netherlands.; ^3^ Institute for Computing and Information Sciences, Radboud University, Nijmegen, the Netherlands.

**Keywords:** decisional needs, shared decision making, stress urinary incontinence, qualitative research, treatment

## Abstract

**Background:**

Choosing a treatment option for female stress urinary incontinence (SUI) is a preference-sensitive decision. Nowadays, shared decision making (SDM) is the preferred way of decision making. SDM considers the needs patients have regarding the decision-making process. The aim of this study was to identify decisional needs of women who are making a treatment decision for SUI.

**Materials and Methods:**

Semistructured interviews were planned with women who had been seeking treatment for SUI. Patients were recruited in two teaching hospitals in the Netherlands. Interviewers used a topic list based on the Ottawa decision support framework. The interviews were transcribed and coded. Themes and subthemes of factors relating to the treatment decision-making process were identified and described.

**Results:**

We interviewed a total of 16 women. Four major themes of SUI patients' needs were identified: information on disorder and treatment, SDM, personalized health care, and consideration for social context. Within these themes, specific needs varied between individuals. In addition to the provision of objective information, other important identified needs were subjective, such as acknowledgment of symptoms and feeling understood by a physician. It was important for patients that they had a sufficient amount of time to make their decision.

**Conclusions:**

To ensure a good quality treatment decision in female SUI, several topics need to be addressed in an SDM process. The themes of decisional needs identified in this study can help improve the decision-making process.

## Introduction

Stress urinary incontinence (SUI) is defined as loss of urine on effort, exertion, sneezing, or coughing.^1^ It is a prevalent condition, affecting more than 20% of women, with a negative impact on quality of life.^2,3^ Choosing a treatment option for female SUI is a preference-sensitive decision for both patients and physicians. Several conservative and surgical options are available each with their specific advantages and disadvantages regarding outcomes and risks.^4–8^

Currently, shared decision making (SDM) is widely regarded as the preferred manner of decision making, especially in conditions where there is not one superior treatment option.^9^ In SDM, patient and physician form a team that discusses the options that are most suitable for the individual patient and then decide together on the type of treatment.^10^ To ensure a high-quality decision-making process, a patient must be ready for SDM and her decisional needs should be addressed. Factors in general that play a role in readiness for SDM are: knowledge of the condition and options, knowledge of SDM, and encouragement by their physician to take part in SDM.^11,12^

In this study, we aim to identify the decisional needs of women with SUI making a treatment decision.

## Materials and Methods

The Consolidated Criteria for Reporting Qualitative Research criteria have been applied to report on the process of acquisition and analysis of our qualitative data.^13^ We have included a completed checklist in the Supplementary File S2.

### Participants and research team

Semistructured interviews were held with Dutch women who were or had been seeking treatment for SUI. We recruited patients in an academic medical center (Radboud University Medical Center, Nijmegen, the Netherlands) and a nonacademic teaching hospital (Gelderse Vallei Hospital, Ede, the Netherlands). Gynecologists in the participating hospitals selected eligible women with SUI based on the corresponding diagnosis code of the International Statistical Classification of Diseases and Related Health Problems 10th Revision. We did not exclude women based on age, comorbidity, prior or pending treatments, and treatment outcomes as we were interested in a diversity of women.

One of the researchers (M.B.E.G. or E.d.S.) verbally contacted the patient for participation in the study. Before the interview, all women received a patient information form and signed an informed consent (IC) form. Participants were not paid for their participation. We included women in two different time periods: 10 women in 2014 and 2015 and 6 women in 2021. In the first period, enrolment continued until data saturation was acquired, which occurred after eight interviews were held.^14,15^ Interview 9 and 10 were performed for data consolidation.

The interviews were processed and analyzed but not yet published. To consolidate our data form the first period and to make sure that no new items had arisen, we initiated a second inclusion period. Furthermore, we aimed to collect more details on the counseling consultation and clarify and deepen items that had surfaced in the first set of interviews. After four women, data saturation was already reached and two more participants were interviewed for data consolidation. A gynecologist and clinical researcher (M.B.E.G.) performed the first 10 interviews and a master student (E.d.S.) performed the last 6 interviews, both female.

M.B.E.G. and E.d.S. were not involved in the counseling and decision-making process of the participants they interviewed. They were trained in the clinical condition and conducting and coding interviews before the start of the study. K.B.K. is an associate professor in urogynecology, MdV is an assistant professor in information and decision sciences. Both are experienced researchers with experience in performing and analyzing qualitative studies.

### Procedure

Depending on participants' preference, the interviews were held face to face in the hospital, by telephone, or screen to screen. During the interview, we used open questions as much as possible. Interviews were planned to last between 30 and 60 minutes. One patient asked to have her partner present during the interview, other interviews were held with only the interviewer and participant. Interviews were audiorecorded and then transcribed. Demographic characteristics of participants were collected. Limited field notes were taken during the interviews. Each interview was discussed afterward with a researcher experienced in qualitative research to process the data and identify important information.

Before start of the interview, a participant was informed about the background of the interviewer and on the purpose of the study. The interviewer explained that she was interested to find out how patients experienced their journey while seeking help for SUI, to improve the information and counseling process for SUI patients and their health care providers in the future. If SUI diagnostics and treatment had not yet been finished, participants were assured that participation in the study would not affect further consultations and treatment.

Concerning the methodological orientation, a pragmatic approach bordering on the grounded theory methodology was applied. The grounded theory method intents to develop a theory “grounded” on the research data.^16^ In this study, we used known information on patients' needs and intended to study the specific needs for women with SUI.

A topic guide for the interviews was developed, based on the Ottawa Decision Support Framework (ODSF)^17^ and on patients' needs in decision making in general.^11,12,18^ The ODSF visualizes the interaction between patients' needs, decision support, and the final treatment choice when there are multiple treatment options with different benefits and disadvantages. It assists health care providers and researchers to assess patients' needs.^17^ The topic guide (Supplementary File S1) included questions on expectations before the consultation, experiences with the consultation regarding information provision, the counseling of treatment options, the decision-making process, and the interaction with the physician. When applicable, questions were asked on expectations and experiences with treatment results and complications and on satisfaction with the outcome after therapy.

Finally, we asked participants to indicate what was most valuable for them in the counseling and decision-making process and what they would alter or had wished to be differently.

The study was ethically approved by the Medical Ethics Committee in both hospitals. IC forms were stored and are only accessible to researchers with granted access (M.B.E.G. and K.B.K). Every participant received a unique code to which the data of the interviews is linked, to ensure patient privacy. Coded data are stored in the digital research environment of the Radboud University Medical Center.

### Analysis

A thematic analysis of the data was performed.^19^ We used an inductive approach in which the themes were derived from the data. During the process, the six phases as described by Braun et al.^20^ were attended to: familiarizing oneself with the data, generating initial codes, searching for and reviewing themes, defining and naming themes, and producing the report. Every interview was analyzed by two or three analysts (M.B.E.G. and one or two master or PhD students). Three different students participated in the open coding process. First, transcripts were read several times to familiarize the analysts with the data. Then, every interview was coded using the software program ATLAS.ti Scientific Software Development GmbH. We started with open coding. When coding of the texts differed, discussion between analysts followed until consensus was reached. The analysis was conducted in the same period as the interviews were held. When new items emerged from analysis, we paid attention to these topics in following interviews to clarify and deepen data. Because the codes did not differ between the two time periods, we analyzed the data from the 16 interviews as one group.

Connections between the codes were identified, with repeatedly going back to the transcripts during this process, creating code groups. Two additional research team members (K.B.K. and M.d.V.) joined M.B.E.G. and E.d.S. in discussions on the codes and code groups to eventually form the final themes and subthemes. When describing the themes, quotes from the interviews were used for illustration.

### Rigor

To describe scientific rigor in qualitative research, Baillie has proposed criteria to evaluate trustworthiness: credibility, dependability, transferability, and confirmability.^21^ In this study, credibility is demonstrated by triangulation (multiple researchers collecting and analyzing data) and peer debriefing after every interview. Dependability is shown by using the same interview topic list in every participant, transferability by including a detailed description of the process of data collection. Lastly, confirmability is shown by using independent interviewers, who were not involved in counseling of the participants.

## Results

Nine participants were interviewed face to face, five by phone and two screen to screen (*n* = 16). Patients' characteristics are described in Table 1. Women talked about accepting their SUI symptoms until they became unmanageable for them, and frequently described one specific moment of urinary leakage after which they decided to seek help for their symptoms. The impact of SUI on their daily lives was described, including embarrassment and shame. Often preventive measures were undertaken during sports and occasionally activities were avoided completely. In this study, we focused on the qualitative analysis of the steps taken after seeking help: patients' needs in the counseling process regarding treatment options for SUI. Four themes of patients' needs were identified: information on SUI and SUI treatment options, SDM, personalized health care, and consideration of their social context. Table 2 shows an overview of themes and subthemes.

**Table 1.** Patient Characteristics

Participant	Age (year)	Center	Treatment	Interview after treatment (yes/no)
1	47	RUMC	MUS	Yes
2	34	RUMC	MUS	Yes
3	45	RUMC	MUS	Yes
4	43	RUMC	PFMT	No
5	63	RUMC	MUS	Yes
6	64	RUMC	MUS	Yes
7	38	RUMC	PFMT	Yes
8	41	RUMC	MUS	No
9	70	RUMC	MUS	Yes
10	47	RUMC	PFMT	Yes
11	57	RUMC	Pessary	Yes
12	58	RUMC	MUS	Yes
13	42	ZGV	MUS	No
14	50	ZGV	MUS	No
15	74	ZGV	MUS	Yes
16	63	RUMC	PFMT	Yes

MUS, midurethral sling; PFMT, pelvic floor muscle therapy; RUMC, Radboud University Hospital; ZGV, Gelderse Vallei Hospital.

**Table 2.** Themes of Patients' Needs

Themes	Subthemes
Information on disorder and treatment	Valuable and missing information Types of information provision
Shared decision making	Empowerment patient Influence of physician Type of counseling
Personalized health care	Counseling based on patients' characteristics Feeling understood Time to contemplate decision
Consideration of social context	Influence of partner or family Influence of peers Influence of media Perceived influence of work

### Information on disorder and treatment

Participants frequently mentioned information regarding the condition itself, effectiveness of treatment, the recovery, complications, and invasiveness of the treatment as important factors in decision making.

Information on SUI was also important to be better able to judge the severity of their symptoms. Obtaining information about the treatment and condition reassured most patients. Eight women described information on the effect of the treatment as main contributor for choosing a specific treatment. This was especially the case in patients choosing midurethral sling (MUS) surgery.

“I think after this [surgery] the problem is solved and I will not accidentally leak urine anymore. For me, that has been the most important factor in making the decision. I just want that [incontinence] to be over with.” (interviewee 14)

Despite the differences in effectiveness, concerns about invasiveness of surgery and possible complications were expressed as a factor that can lead to refrainment from treatment or to choosing a more conservative treatment.

“I prefer not to have an operation when it is not necessary, not to have an implant in my body when it is not necessary.” (interviewee 16)

Women wanted information on recovery and possible complications of surgery. In addition, the time period it takes to reach maximum treatment effect was an important factor, especially when considering pelvic floor muscle therapy. Multiple women expressed a fear of complications after surgery. As a result, women sometimes preferred nonsurgical treatment options.

“A recovery period in the hospital after surgery, that is not for me. I will start physiotherapy.” (interviewee 12)

In general, women valued the information provision and physical education by a pelvic floor therapist.

Question (Q): “Did your physiotherapist explain how the pelvic floor works? … And did you find that useful?” Answer (A): “Yes, definitely, definitely.” (interviewee 7)

Patients valued the information provision by their physician during the consultation itself. They also found it helpful to have another source of information which could be studied at home.

“… but after a while [during the consultation] I was pretty much exhausted so I could not take in more information. It would be better if it [patient information] was also available in a patient leaflet, so I could have read it again afterward.” (interviewee 4)

Another subject that arose from the interviews multiple times, was the need for information on sexuality in relation to SUI. In retrospect, participants had frequently missed getting this information. Women wondered if pretreatment coital urine loss was associated with SUI, and there was a need for information on how different treatments can have an effect on sexuality, in a positive or negative way.

“I thought it was remarkable that intercourse, having intercourse, was not discussed during the consultation. … It is very essential of course and it can also be a significant factor in the decision-making process. If I would have had urinary incontinence during intercourse after treatment that would have been awful to me.” (interviewee 3)

Looking back, several women expressed they had received insufficient or incorrect information, not only on topics such as sexuality and recovery or complications after surgery, but also more in general on advantages and disadvantages of the different options.

“He [refers to the gynecologist] talked about a week [of recovery] in total and that was definitely not true. Because after the operation, I was in a lot of pain, really a lot of pain.” (interviewee 2)

Q: “And, in hind sight, what would have made it [the decision-making process] different for you?” A: “Well, I think, I would have liked to hear more options. And to know the advantages and disadvantages of the options.” (interviewee 9)

### Shared decision making

The majority of the interviewed patients reported that they felt able to take an active part in the SDM process and they felt they could make a decision supported by their physician.

“He [general practitioner (GP)] knew about my medical history and said: “I can perform a physical exam myself, but you are so obvious in what you want [refers to MUS surgery].” (interviewee 12)

Some women reported that their physician did not involve them in the decision-making process. Physicians making a treatment decision themselves with the best interest of the patient in mind is called paternalistic decision making.^22,23^ While some women did not mind the paternalistic decision making because they trusted the expertise of their physician, other women felt like they did not have a choice. Either their physician only discussed a single treatment option or it was evident that the treatment preferred by the physician was regarded as the only feasible option. One participant expressed she felt pressured toward MUS surgery and that the treatment decision was solely made by the physician himself.

Q: “And what was your opinion of the treatment choice, did you believe that you could make that decision yourself?” A: “No, no, I was overwhelmed. I was really overwhelmed.” (interviewee 9)

Some participants already knew what treatment they preferred before the consultation, while other women did not know which treatment options were possible before consulting a physician.

### Personalized health care

The majority of patients reported they felt heard or understood by their physician at some point during the decision-making process. A physicians' acknowledgment of SUI symptoms and its effect on daily life made it easier for women to progress to making a treatment decision. It also helped women to feel more empowered to participate in an SDM process.

“Acknowledgment of my symptoms is the most valuable [element of the consultation] that I have experienced actually. I felt that there was genuine attention for my problem.” (interviewee 14)

In case a woman did not feel heard or understood, this negatively affected the perceived quality of the decision-making process.

“… you know, at a certain moment I thought: You [refers to the physician] don't understand me, so never mind.” (Interviewee 14)

Some women felt less at ease going to a consultation when they knew that they would encounter a male physician. Being counseled by a male physician attributed to not feeling understood in some women.

Counseling based on individual patient characteristics is part of the SDM process. Characteristics include patients' preferences and values but also physical factors. Some participants described that their obesity played a role in the suggested treatment options: their physicians were not keen to recommend MUS surgery but instead advised to lose weight first. This felt frustrating, made them feel hopeless, and gave them the feeling of not being heard.

“I am obese and I am always easily under the impression that a GP believes that my obesity is associated with the complaint. So no, I have felt for a while that I was sent from pillar to post. (interviewee 14)

Another woman was diagnosed with a combination of SUI and an underactive bladder. The option of MUS surgery was discussed with a higher risk for self-catheterization. Therefore, she renounced the operation.

Multiple women stated that they were given enough time to make a treatment decision. Several women reported to have made the decision during the consultation itself and to their satisfaction. However, a couple of women felt they had not had enough time to consider the treatment options. One participant even felt pressured to consent.

Q: “Did you feel that you had to decide at that moment or was there time to think it over?” A: “No, I had to decide on the spot.” (interviewee 9)

### Consideration of social context

Most women reported that they had sought confirmation and support of their family or partner when deciding for SUI treatment. Most partners supported women in the decision-making process. Although participants discussed their SUI symptoms with their partner or family, most women made the treatment decision by themselves.

Q: “Did you consult others on your treatment decision?” A: “Yes, my husband, he agreed with me that something needed to be done.” (interviewee 15)

Women also took the impact on their family into consideration when making a choice for a treatment option. Recovery of surgery was reported to be an important issue to discuss with family, since there are restrictions after surgery and women are dependent on others to care for them and their families. One woman had postponed MUS surgery in the past because of not being able to take care for her small children after the operation.

“In the beginning, when the children were young, I thought: I don't know how I would manage this [surgical treatment]. Four to six weeks not being able to take care of my family… So I took that into account in making my treatment decision at the time.” (interviewee 1)

Some women also discussed SUI treatment with friends, usually when they had the same symptoms. In most cases, friends and peers were supportive of women making a treatment decision.

“A friend influenced my treatment decision… she also had pelvic floor problems. And we talked about it, that it would be really nice if the symptoms would disappear.” (interviewee 3)

Experiences of women who already had undergone SUI treatment sometimes influenced the treatment decision of women still having to choose.

“Somebody told me that it [the recovery after surgery] was really difficult and unpleasant; that you cannot pee and those sort of things. She considered it to be really challenging.” (interviewee 15)

Another component influencing women's decision emerging from the interviews, was the influence of the media. One woman mentioned that she watched a TV program on SUI and treatment options, which gave her more insight into the treatment options. Another participant had read articles on SUI and SUI treatment in the newspaper. Other women reported that they were influenced by negative media attention on the use of vaginal mesh for pelvic organ prolapse.

“I have a tape [midurethral sling]; it is a foreign object in my body. Especially at the time that there was so much information on vaginal mesh… I was worried.”(interviewee 9)

When considering MUS surgery, several women felt like being a burden toward their employer and coworkers because of the recovery period after the surgery. The elective character of MUS surgery amplified this feeling. However, none of the women reported to have discussed these considerations with their employer.

Q: “So the recovery period [after surgery] would have been an obstacle for you?”

A: “Yes, the recovery period, to take time off and bother my colleagues with not being able to work for a certain timeframe, because of my decision... Now I am going to have this surgery performed because I want to. It sounds silly.” (interviewee 3)

## Discussion

In this study, we have identified patients' needs in counseling conservative and surgical treatment options for SUI in women. The identified patients' needs can be grouped into four themes: information provision, SDM, personalized health care, and consideration for personal context. Our results are in line with previous studies on female SUI patients showing that multiple factors play a role in the decision-making process and that a treatment choice is not only based on objective information and physical characteristics.^24,25^ Furthermore, most women had specific, individual needs within the different themes we have identified. As a reason to seek help for SUI symptoms, most women described a point of no return, a moment in which the SUI symptoms became unacceptable for them. This was also shown by a study by Lynch et al.^26^

### Information provision

Having good-quality information on the condition and treatment options was deemed essential to be able to participate in SDM. Patients receiving adequate information are better suited to make a treatment decision, better equipped to engage in SDM, and have a more realistic expectation of treatment outcomes.^9,11^ Women missed getting information on sexuality in relation to SUI and the treatment options. Participants experienced that physicians found it difficult to discuss the subject of sexuality, while patients feel a need to know what effect the condition and treatment option can have on their sex life.

### Shared decision making

Patients felt empowered and better equipped to participate in SDM when their physical symptoms were acknowledged and when they felt their physician understood the effect of their symptoms on daily life. This is also reported in the study by Lynch et al.^26^ Not every patient is ready for SDM at any given moment. A physician can support a patient to discover her needs in the decision-making process and provide in those needs where possible.^12,17,27^ Patients can find it hard to speak up for themselves even when they are well educated and informed or when they experience a power imbalance in the patient/physician relationship.^25,28^ When patients' decisional needs are met, the quality of the resulting decision is higher, which can result in better implementation of the treatment by the patient and also stimulate appropriate use of health services.^17,27,29^ Ong et al. tested a decision aid for surgical treatment options in women with SUI to support SDM. It was a small sample of women but they reported a favorable outcome regarding the experienced decisional conflict.^30^ Some women did not mind paternalistic decision making.

Others retrospectively felt that they were not involved enough in the decision-making process and wished they were given more information and guidance in this process by their physician. Patients want to be asked about their treatment preferences and their preferred level of participation in the decision-making process.^31^ There can be several different reasons why physicians do not engage in SDM. Some physicians are still skeptical of SDM and its positive effects on patient outcome and satisfaction, but also because of perceived negative effects on busy schedules, time restraints and having to change their way of working.^32^ Others assume that a certain patient is not able to take part in SDM and do not try to engage this patient in the process. Other physicians think they practice SDM but in fact practice paternalistic decision making.^22^

### Personalized health care

Acknowledgment of symptoms and feeling understood were perceived as important factors for patients to feel at ease and comfortable during the consultation. When patients have a good relationship with their physician, it is easier to engage in a more equal relationship in which it is possible to ask questions and have discussions on treatment options.^11,12,24^ Some participants experienced an uneasiness, before and during the consultation, which they attributed to the male gender of the physician. In a study simulating counseling consultations in women with pelvic floor problems, patients perceived higher quality SDM with a female doctor.^33^

Limited time spent with a physician can result in not being able to build up a good patient/physician relationship, insufficient information provision, not enough time to process information, and make a good quality decision.^12,34^ The amount of time a patient needed to decide was individually determined. Some patients had informed themselves before the consultation and already had thought of or decided on treatment options, whereas others only started pondering options after the consultation.

Written information can be essential so that patients are able to read this at home or to schedule a subsequent consultation.

### Consideration of social context

Women valued opinions and experiences of peers with SUI and opinions of the partner, family, and friends were also considered by patients in the decision-making process. This confirms earlier research.^26,35,36^ Also, working conditions played a role in decision making, when women were considering the strain of treatment on their work and colleagues.

### Strengths and limitations

The strengths of this study include the participation of different interviewers and researchers. There were frequent discussions on data collection and analysis, which is known as researcher triangulation.^37^ It ensures more objective opinions on the data collection, coding, and interpretation of data. Data saturation was leading to determine the number of inclusions. During the recruitment and interviewing process, we transcribed and analyzed the data on a rolling basis. This made it possible to further explore interesting items in subsequent interviews that had surfaced earlier on. In the second recruitment period for example we more specifically collected data on the counseling consultation. One limitation of this study is that four participants had not yet undergone treatment at the time of the interview and we did not repeat the interview after treatment. Women may have had different feelings or recollection of counseling after the treatment depending on outcome, resulting in recollection bias. Another limitation is that we do not know the characteristics of the women who declined to participate in the interviews, nor their reasons to decline, which could have led to selection bias.

## Conclusions

Information provision, SDM, personalized health care, and influence of others are the themes we have identified as patients' needs in counseling treatment options in female SUI. It is important to explore the specific needs from each theme in every individual patient.

### Implications for the future

SDM can be improved by using decision supporting tools, such as a patient decision aid. Our findings can be used to tailor such tools to patients' needs. Furthermore, it would be interesting to see if the identified themes in patients' needs also apply to other urogynecological symptoms, such as pelvic organ prolapse. Finally, future research could evaluate whether patients' satisfaction with the decision-making process and the outcome of the treatment increases when the identified needs are taken into account.

## Supplementary Material

Supplemental data

Supplemental data

## Abbreviations Used

IC: informed consent
MUS: midurethral sling
ODSF: Ottawa Decision Support Framework
PFMT: pelvic floor muscle therapy
RUMC: Radboud University Hospital
SDM: shared decision making
SUI: stress urinary incontinence
ZGV: Gelderse Vallei Hospital

## Appendix A1: Suggestions to Apply in Daily Practice

- Provide evidence-based information in different forms (*e.g.*, verbally, leaflets, website, decision aid), suited to the individual needs of a patient. Ask whether the patient has understood the information.
- Acknowledge the stress urinary incontinence symptoms and the impact they have on the patients' daily life.
- Stimulate the patient to explore her values regarding treatment options and invite her to take part in a shared decision-making process.
- Discuss the impact of social context, such as family, work, peers or media, on making a treatment decision.
