# Supplementary material for: Identifying Womens' Needs in Making a Treatment Decision for Stress Urinary Incontinence: A Qualitative Study
Source: Womens Health Rep (New Rochelle). 2023 Jul 18;4(1):358–66. doi: 10.1089/whr.2023.0002 (PMC10354721; doi:10.1089/whr.2023.0002)
Supplement: Supplemental data [file Suppl_FileS1.docx]

Supplemental Digital Content 1: topic guide for interviews

*Before the consultation with gynecologist or urologist:*

What were your expectations and experiences regarding the consultation with the physician who referred you to the gynecologist/urologist? On topics of:

- Anamnesis, physical examination
- Additional tests (for example test of urine sample)
- Information on urinary incontinence (received before the consultation in hospital, gotten during consultation with referring physician)
- Possible treatment options, and if so which? Already counseling or opinion of referring physician, information received verbally, in writing, internet?
- Where you offered a treatment option by the referring physician?
- What was your experience of this consultation? What did you value, what did you miss, what would you like to see differently?
- Did you feel taken seriously?
- Was there enough time during consultation?
- Was it possible to ask questions?

*The consultation with gynecologist or urologist*

What were your expectations and experiences regarding the consultation with the gynecologist or urologist? On topics of:

- Anamnesis, physical examination (effect on daily life, for example sexuality, sport, work, hobbies)
- Additional tests (for example test of urine sample, flowmetry)
- Information on urinary incontinence
- Possible treatment options, which ones were named? What was named: (dis)advantages, side effects, complications, chances of success, time to optimal result, possibility to transfer to another treatment? Way of counseling: directive, open, individual? Information received verbally, in writing, internet?
- What was your experience of this consultation? What did you value, what did you miss, what would you like to see differently?
- Did you feel taken seriously?
- Was there enough time during consultation?
- Was it possible to ask questions?

*Making a treatment decision*

- What was your experience of the decision making process?
- Have your values regarding therapy been discussed (effect, investment, (non)invasive, time line)?
- What were your treatment values?
- Which factors have played a role in making a treatment decision (also non-medical such as health insurance, experience of peer)
- Did you consult others before you made a decision (family, friends, general practitioner)?
- When did you make the decision (before, during, after consultation)? Did you get time to contemplate the decision? Could you ask additional questions and how (for example second consultation)?
- Who made the treatment decision (you, the physician, family of friends, or together)?
- Did the ultimate decision fit your situation?
- Were you content with the way the treatment decision was made?

*After therapy*

What were your expectations and experiences regarding the chosen therapy?

- Time to treatment effect, which effect?
- Was the effect or recovery according to the given information? If no, did you expected it to be so?
- Did you switch to another form of treatment?
- How content are you in hind side with the treatment decision? What are reason that you are (not) content?
- When would you not have been satisfied?

*Concluding*

- What did you find most valuable in the counseling and decision making process?
- What would you have liked to be different?

Which three outcome measures are most important for you in a treatment (for example success percentage, percentage content)?
